# Utilization of PFGE as a Powerful Discriminative Tool for the Investigation of Genetic Diversity among MRSA Strains

**Published:** 2017-03

**Authors:** Solmaz OHADIAN MOGHADAM, Mohammad Reza POURMAND, Masoumeh DOURAGHI, Samira SABZI, Parisa GHAFFARI

**Affiliations:** 1. Uro-oncology Research Center, Tehran University of Medical Sciences, Tehran, Iran; 2. Dept. of Pathobiology, School of Public Health, Tehran University of Medical Sciences, Tehran, Iran; 3. Biotechnology Research Center, Tehran University of Medical Sciences, Tehran, Iran

**Keywords:** Molecular typing, Methicillin-resistant *staphylococcus aureus*, Pulsed-field gel electrophoresis, Burn units

## Abstract

**Background:**

Methicillin-resistant *Staphylococcus aureus* (MRSA) is a significant challenge to the burn patient. The implementation of proper monitoring programs and prompt identification of epidemic MRSA strains are critical to consequently control and eradicate potential outbreaks. This study aimed to define the genetic relatedness of MRSA strains isolated from burn patients by analyzing the large fragments of DNA.

**Methods:**

In this cross-sectional study, 126 pus/wound swabs from skin and soft tissue infections (SSTIs) were collected from inpatients of Shahid Motahari Burn Center (Tehran, Iran) in 2013. Then, molecular typing of MRSA was achieved by Pulsed-Field Gel Electrophoresis (PFGE).

**Results:**

The PFGE analysis of MRSA indicated 31 single types and 5 common types. There was a significant diversity in the chromosomal digestion pattern of the MRSA strains explained by the acquisition of MRSA from various sources.

**Conclusion:**

The permanent import of novel types of MRSA strains despite the rigorous infection control measures carried out within the center. The importance of PFGE in understanding the epidemiology of MRSA may serve as a basis for the development of rational control strategies.

## Introduction

*Staphylococcus aureus* is one of the most common and important pathogens responsible for burn wound infections (1). While burns are considered one of the most serious forms of trauma (2), burn injury remains to be a major public health concern and a leading cause of morbidity and mortality all over the world (3). Nonetheless, the surge of methicillin-resistant *S. aureus* (MRSA) outbreaks has caused a major setback in nosocomial infections. Moreover, these strains can produce invasive infections, especially in vulnerable patients (4).

Some MRSA strains are epidemic and disseminate quickly. Therefore, the implementation of prompt monitoring programs is essential for the rapid identification of the prevailing type of bacteria in order to control the spread of resistant strains and eradicate sequential outbreaks. In addition, the typing of MRSA isolates is extremely valuable for the execution of proper infection control measures (5). Presently, there has been a breakthrough in the typing methods based on genotypic characteristics of bacteria (6).

Pulsed-field gel electrophoresis (PFGE) is the most common tool for molecular typing and it is the method of choice for DNA fingerprinting of MRSA and other pathogens (7). Consequently, burn centers should routinely explore the particular pattern of burn wound microbial colonization and their genetic diversity, antibiotic susceptibility profiles of pathogens involved in nosocomial infections and their tendency to spread in the hospital setting.

Until now, there has been no study regarding the molecular characteristics of MRSA collected from burn patients in Iran. This study aimed to define the genetic relatedness of MRSA strains isolated from burn patients by analyzing the large fragments of DNA.

## Methods

### Setting

This study was conducted at the Shahid Motahari Burn Center, the largest burn center in Iran affiliated with Iran University of Medical sciences (IUMS), Tehran, Iran. This center consists of three separate units: men, women, and children.

### Study design

In this cross-sectional study, 126 pus/wound swabs from skin and soft tissue infections (SSTIs) were collected from inpatients of Shahid Motahari Burn Center over a 6-month period in 2013. Only one isolate per patient was involved in this study.

### Ethical Issues

The Ethics Committee of Tehran University of Medical Sciences approved the study protocol, which was conducted in accord with the tenets of the Helsinki Declaration. All participants signed a written informed consent.

### Bacterial isolates

After sampling, the swabs were immediately transferred to a transport medium, and sent to Tehran University of Medical Sciences (TUMS) molecular Microbiology laboratory, where they were subcultured onto a blood agar and incubated overnight at 37 °C. Subsequently, *S. aureus* was then identified using confirmatory tests (Gram’s stain, catalase, coagulase and DNase tests and mannitol fermentation on mannitol salt agar (MSA)).

### Confirmation of methicillin-resistant *S. aureus*

Phenotypic method: Bacterial suspensions of all *S. aureus* isolates equivalent to 0.5 McFarland were prepared and cultured on Mueller-Hinton agar medium plates containing 4% NaCl and an oxacillin disk (1 μg oxacillin; MAST Diagnostics, Merseyside, U.K.), incubated overnight at 35 °C. The growth inhibition zones were interpreted by the Clinical and Laboratory Standards Institute (CLSI) guidelines (8) thus; isolates were accordingly screened for methicillin-resistance.

### Genotypic method

Bacterial DNA was extracted using a Dneasy kit (Qiagen, Valencia, CA) and according to the manufacturer’s protocol; lysostaphin enzyme (5 mM) was used in the first stage. The *mecA* gene was then detected in DNA extracts using PCR assay (9).

### Pulsed-field gel electrophoresis

The MRSA isolates were genotyped by PFGE based on the previously described protocol (10) with some modifications. Approximately, 10 to 20 *S. aureus* colonies previously incubated for 24 h were inoculated into 10 ml of tryptic soy broth (TSB) and were incubated overnight at 37 °C. Broth cultures were centrifuged at 1500×g for 20 min, the supernatants were discarded and the pellets were resuspended in sterile saline and transferred to a pre-weighed 1.5 ml microcentrifuge tube, centrifuged again at 13000 × g for 20 sec removing the saline as much as possible. Next, the pellet was weighed and an equivalent amount of sterile saline was added. A tube of 2% agarose was melted and transferred to a 58 °C water bath. Then, 30 μl of cell suspension was added to 400 μl of EC buffer (6 mM Tris, 1 M NaCl, 0.1 M EDTA, 0.5% Brij 58, 0.2% deoxycholate and 0.5% Sarkosyl).

Then, lysostaphin (10 mg/ml) and 450 μl of 2% agarose (58 °C) were added and gently mixed and immediately dispensed into a small disposable plug mold and cooled for 30 min. The plugs were removed from the molds and introduced into a lysozyme (20 mg/ml) containing EC buffer and incubated at 37 °C for 5 h and the blocks covered with an ES buffer (0.4 M EDTA, 1% Sarkosyl) containing Proteinase K (20 mg/ml) were incubated overnight in a 50 °C water bath. According to the following procedure, the plugs were washed with CHEF-TE buffer (0.1 M Tris PH7.5, 0.1 M EDTA): The initial wash required no incubation time and for three washes, the washing step was repeated at 30 min intervals. The plugs were stored at 4 °C.

For the DNA restriction digestion step, the plugs were washed twice in a DNS buffer (0.1 M Tris PH8.0, 5 mM MgCl_2_), each time for 30 min. Then, DNA digestion was performed using a 30 IU *Sma*I (Roche Diagnostics) for 16 h at 25 °C. The DNA macrorestriction fragments were separated in the agarose gels by PFGE using a contour-clamped homogeneous electric field apparatus, i.e. the CHEF DRIII System (Bio-Rad, Hercules, California, USA) on a 1% agarose gel prepared in a 0.5 M TBE (Tris-borate-EDTA) buffer. The running parameters were as follows: strength 6 V/m; temperature 12 °C; pulse time increasing from 5 to 50 sec; and duration 22 h; 14 °C; 0.5 M TBE as running buffer. The *Salmonella enterica* serotype Braenderup H9812 (CDC), digested with 20 IU *Xba*I (Roche Diagnostics) for 20 h at 37 °C was used as a molecular size marker and included in each gel.

After the completion of the electrophoresis run, the gel was stained in 1μg/cm of ethidium bromide solution for 20 min. Finally, the gel was photographed under UV transillumination. Digital images stored electronically as TIFF files were analyzed with the GelCompar software (Applied Maths, Belgium) by using the Dice correlation coefficient and the UPGMA method (unweighted pair group method using arithmetic averages). Clones were defined according to a similarity (Dice) coefficient greater than 0.8.

## Results

### MRSA distribution

*S. aureus* was isolated in 57 (45.24%) of the 126 pus/wound swabs taken from the skin or soft tissue infections, SSTIs. By using phenotypic (disc diffusion method) and genotypic (PCR for detection of *mecA* gene) methods, 43 (75.43%) of the isolates were confirmed as MRSA.

### Molecular typing

**Fig. 1:**
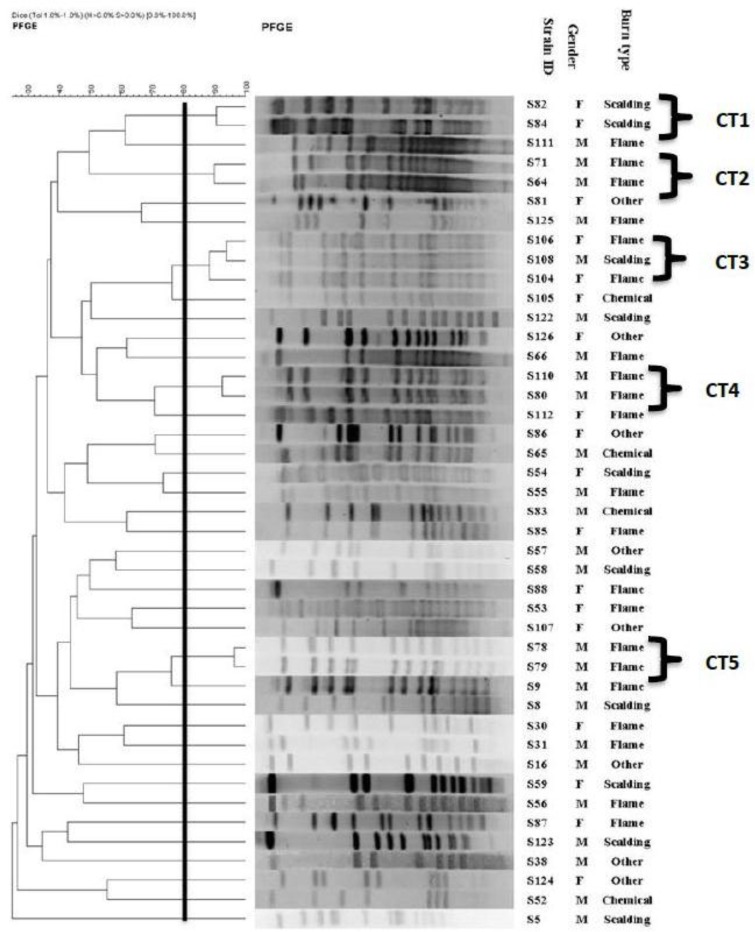
Dendrogram based on the relationships among MRSA isolates derived from the UPGMA and Dice coefficients, using Gel ComparII software

All MRSA isolates were analyzed by PFGE. In this study, 5 common types (CT) and 31 single types (ST) were observed (ST and CT represent the dissimilar and pulsotypes with a minimum similarity of 80%, respectively) among the 43 of the MRSA isolates (Fig. 1). According to the cluster analysis of MRSA strains, majority of the CTs were isolated during February.

## Discussion

We used PFGE as a powerful discriminative tool to investigate the epidemiological characteristics of MRSA isolates. The chromosomal digestion patterns allowed us to differentiate the MRSA isolates, even the ones demonstrating the same antimicrobial susceptibility pattern. Genotyping methods such as PFGE may lead to a better insight of MRSA transmission and therefore help determine appropriate control and preventive measures. PFGE is a valuable technique for the characterization of outbreaks and their sources (11–14). For example, we found 5 CTs in our study, which represented five outbreaks during different periods. All the isolates belonging to the CTs had a similar antibiotic resistance pattern. Dates of the isolation show that some isolates, having similar pulsotypes, were isolated within three consecutive months (CT3 in Apr, Jun, and Jul). Two of the CT (CT2 and CT4) were isolated simultaneously, in Feb. Moreover, according to the collected information, MRSA clustered in CT3 isolated from patients with a history of hospitalization suggest the consideration of a source of infection outside the hospital.

Apparently the inter-hospital spread of MRSA strains had occurred. On the other hand, the simultaneous isolation of a CT (CT3) from different wards of a hospital (men and women) possibly suggests the existence of a common source of infection, such as infected patients or contaminated equipment. In the present study, enzymatic digestion with *SmaI* revealed a high diversity of the digestion profile i.e. 31 single restriction pattern types for 43 MRSA isolates. The significant diversity of MRSA could be explained by acquisition of MRSA from various sources. In addition, colonized patients could potentially spread these strains.

This occurred despite all the strict strategies, such as the regular decontamination of all hospital surfaces, topical application of silver sulphadiazine and mupirocin, routine washing of the burn surface areas and preventive measures, including regular screenings of all bacteria isolated from burn wounds. The significant role the environment plays as a ground for outbreaks has also been reported in other studies (15). The overall proportions of MRSA contamination in non-hospital environments were high and there is the risk MRSA strains cross-transmission among the population, so recognize further the efficiency of the sterilization processes in a non-hospital environment, that relevant department can take measures to progress disinfection of MRSA in non-hospital environments (16).

Thus, this emphasizes the importance of routine molecular typing and eradication of the causative agents to prevent sequential outbreaks, especially in burn centers (17). The PFGE pattern’s database derived in this study can be used to compare future outbreaks as a means to simplify control programs. Our study had several limitations, such as the lack of previous knowledge on the genetic composition of environmental strains for comparison purposes. Additional studies from other regions of the country are required to reach a broader perspective on the clonal dynamics of MRSA contaminating hospitals in Iran.

## Conclusion

We documented a high diversity in the chromosomal digestion pattern of the MRSA strains presented in this study. This result may indicate the permanent import of novel types of MRSA strains despite the rigorous infection control measures carried out within the center. Moreover, the persistence of a certain clone shows the successful adaptation of the clone into the environment due to multiple selection forces, such as antibiotic resistance.

## Ethical considerations

Ethical issues (Including plagiarism, informed consent, misconduct, data fabrication and/or falsification, double publication and/or submission, redundancy, etc.) have been completely observed by the authors.
